# Mitochondrial Proteome Reveals Metabolic Tuning by Restricted Insulin Signaling to Promote Longevity in *Caenorhabditis elegans*

**DOI:** 10.3390/biology14030279

**Published:** 2025-03-09

**Authors:** Xuanxuan Guo, Jiuwei Lu, Long Miao, Enzhi Shen

**Affiliations:** 1 School of Medicine, Zhejiang University, Hangzhou 310058, China; 11963135@zju.edu.cn; 2 Key Laboratory of Growth Regulation and Translational Research of Zhejiang Province, School of Life Sciences, Westlake University, Hangzhou 310024, China; 3 Westlake Laboratory of Life Sciences and Biomedicine, Hangzhou 310024, China; 4 Department of Biochemistry, University of California Riverside, Riverside, CA 92521, USA; jiuwei@ucr.edu; 5 Key Laboratory of RNA Biology, CAS Center for Excellence in Biomacromolecules, Institute of Biophysics, Chinese Academy of Sciences, Beijing 100101, China; lmiao@ibp.ac.cn; 6 University of Chinese Academy of Sciences, Beijing 100049, China; 7 Center for Biological Imaging, Core Facilities for Protein Science, Institute of Biophysics, Chinese Academy of Sciences, Beijing 100101, China; 8 MOE Key Laboratory of Cell Proliferation and Regulation Biology, College of Life Sciences, Beijing Normal University, Beijing 100875, China; 9 Institute of Biology, Westlake Institute for Advanced Study, Hangzhou 310024, China

**Keywords:** aging, DAF-2, mitochondria, quantitative proteomics, metabolism

## Abstract

Aging is a time-dependent process of functional decline influenced by genetic and environmental factors. Age-related mitochondrial changes remain incompletely understood. Here, we found that compared to the wild type, the mitochondria of long-lived *daf-2 C. elegans* maintain youthful morphology and function. Through quantitative proteomic analysis on isolated mitochondria, we identified 257 differentially expressed candidates. Analysis of these changed mitochondrial proteins reveals a significant upregulation of five key mitochondrial metabolic pathways in *daf-2* mutants, including branched-chain amino acids (BCAA), reactive oxygen species (ROS), propionate, β-alanine, and fatty acids (FA), all of which are related to *daf-2*-mediated longevity. In addition, mitochondrial ribosome protein abundance slightly decreased in *daf-2* mutants. A mild reduction in mitochondrial elongation factor G (*gfm-1*) by RNAi extends the lifespan of wild type while decreasing lipid metabolic process and cytoplasmic fatty acid metabolism, suggesting that proper inhibition of mitochondrial translation activity might be important for lifespan extension. Overall, our findings indicate that mitochondrial metabolic modulation contributes to the longevity of *daf-2* mutants and further highlights the crucial role of mitochondria in aging.

## Simple Summary

Mitochondrial dysfunction is intricately linked to the progressive decline of physiological functions during aging. *Caenorhabditis elegans* harboring a mutation in the insulin receptor-like gene *daf-2* exhibit a lifespan approximately double that of the wild type. Quantitative proteomic profiling of isolated mitochondria from *daf-2* mutants has revealed numerous differentially expressed proteins. The identified proteins suggest an upregulation of mitochondrial intermediate metabolic pathways and a concurrent downregulation of mitochondrial translation activity. Lifespan assays confirm that enhanced mitochondrial intermediate metabolic activity significantly contributes to the prolonged lifespan observed in *daf-2* mutants. Moderately attenuating mitochondrial translation activity in wild-type organisms can promote lifespan extension through its influence on lipid metabolism. These findings suggest that mitochondrial metabolic modulation contributes to the longevity of *daf-2* mutants, further highlighting the crucial role of mitochondria in aging.

## 1. Introduction

Aging is characterized by the progressive functional decline of organisms over time [1]. Several hallmarks of aging have been identified, including DNA instability, telomere attrition, cellular senescence, impaired macroautophagy, chronic inflammation, and mitochondrial dysfunction [2]. Mitochondria, which are membrane-bound intracellular organelles, generate chemical energy that drives various biological and pathological processes [3,4]. In recent decades, the pivotal role of mitochondria in aging has been underscored, particularly through Harman’s mitochondrial free radical theory of aging (MFRTA) [5]. This theory posits that mitochondria, as the primary generators of free radicals, substantially contribute to the aging process by promoting oxidative damage. Increasing empirical evidence supports these theories, underscoring the role of mitochondria in aging. Indeed, research has demonstrated that overexpression of mitochondrial catalase extends lifespan in mice [6,7]. Additionally, mutations or knockdowns of certain electron transport chain (ETC) genes, such as *atp-5* and *clk-1*, have been found to lengthen the lifespans of worms and flies [8]. Given the importance of mitochondria in aging, understanding how mitochondrial function is regulated in the context of lifespan extension in *C. elegans* provides key insights into the aging process.

The insulin/insulin-like growth factor-1 (IGF-1) signaling pathway (IIS) is evolutionarily conserved across various species, from yeast to *Caenorhabditis elegans*, *Drosophila*, and mice [9,10,11,12]. Three decades ago, a landmark discovery demonstrated that a single-gene mutation in *daf-2*, a homolog of the insulin or IGF-1 receptor, could double the lifespan of *C. elegans* relative to wild-type (WT) counterparts [13,14]. Subsequent studies confirmed lifespan extension across multiple species with *daf-2* homolog mutations. DAF-16 modulates downstream gene expression to exert its effects on lifespan. Importantly, mutations in *daf-16*, part of the FOXO family of transcription factors, can entirely negate the extended lifespan phenotype observed in *daf-2* mutants [15,16]. Previous studies have demonstrated mitochondrial metabolic abnormalities associated with IIS-mediated alterations in aerobic energy production [17], as well as the preservation of mitochondrial network morphology in intestinal and muscle tissues [18,19]. The potential significance of mitochondrial function in aging prompted us to further explore how mitochondria contribute to the extended lifespan of the *daf-2* mutant. Several key studies on aging in *C. elegans* have identified changes in mitochondrial protein levels. For instance, Walther et al. (2015) found that mitochondrial protein levels decrease with age, especially the ratio of mitochondrial to cytosolic ribosomal proteins, which is less affected [20]. Similarly, Narayan et al. (2016) showed that fatty acid metabolism-related proteins accumulate with aging [21]. These findings suggest that mitochondria play a critical role in *daf-2*-induced longevity. In our study, we investigated changes in the mitochondrial proteome of *daf-2* mutants, focusing on the quantitative analysis of protein changes in various metabolic pathways to explore their roles and mechanisms in aging.

To assess changes in mitochondrial protein levels in *daf-2* mutants, we employed quantitative proteomics. Three common approaches are available: ^15^N labeling, SILAC, and label-free quantification. SILAC uses isotope-labeled amino acids to tag samples, offering high precision but at a relatively high cost [22]. In contrast, label-free methods require no additional labeling but tend to be less accurate and are more susceptible to variations stemming from sample complexity and fluctuations in protein expression [23]. In addition, ^15^N labeling is one of the earliest-developed metabolic labeling methods and offers a simple, cost-effective alternative [24]. Although ^15^N labeling may lead to complex mass shifts in peptides and variations in ionization efficiency among different peptides, these challenges are addressed by employing data correction strategies via the pQuant software developed by Chao et al. [25]. Moreover, the rigorous FDR control and quality filtering methods proposed by Tabb et al. (DTASelect 2.0) [26], which set stringent mass windows and deviation thresholds for both precursor and fragment ions, further ensure the accuracy and reliability of the data. Overall, considering the operational simplicity, cost-effectiveness, and broad sample applicability of ^15^N labeling, we ultimately selected this method for our study.

Here, we investigated mitochondrial functions and the proteome in long-lived *daf-2* mutants compared to WT *C. elegans*. First, we observed that *daf-2* mutants exhibit a slower age-dependent decline in both mitochondrial morphology and function. Quantitative mitochondrial proteomics identified a total of 2425 proteins, of which 257 were differentially expressed in the *daf-2* mutant compared to WT, and most of the expression differences were dependent on DAF-16 activity that is required for the longevity of *daf-2* mutants. Analysis of these changed mitochondrial proteins revealed a notable upregulation of five key mitochondrial metabolic pathways, including branch-chain amino acid (BCAA), reactive oxygen species (ROS), propionate, beta-alanine, and fatty acid (FA) in *daf-2* mutants, which are implicated in *daf-2* longevity. RNAi knockdown of metabolic enzymes (CPT-2, ECH-4, GTA-1) decreases the lifespan of *daf-2* mutants. In addition, we observed a mild reduction in mitochondrial ribosome protein abundance in *daf-2* mutants. Proper suppression of mitochondrial translation activity might be involved in lifespan extension via the downregulation of lipid metabolism processes. Collectively, this study profiles mitochondrial proteome changes in the long-lived *daf-2* mutant, revealing the importance of mitochondrial metabolic tuning in the longevity of *daf-2* mutants.

## 2. Materials and Methods

### 2.1. Strains and Maintenance

All the *C. elegans* strains were cultured at 20 °C on nematode growth medium (NGM) plates and seeded with *E. coli* OP50 following established protocols [27]. The following strains were used in this study: CF1041: *daf-2(e1370)III*; MDQ857: *daf-2(e1370)III*; *daf-16(mu86)I*; MDQ752: *him-8(e1489)IV*; *hqls181[Psdhb-1::mtLS::GFP*; *pRF4(rol-6)](hqls181)*; MDQ772: *daf-2(e1370)III*; *hqls181[Psdhb-1::mtLS::GFP*; *pRF4(rol-6)](hqls181)*; *him-8(e1489)IV*; MDQ809: *daf-16(mu86)I*; *daf-2(e1370)III*; *hqls181[Psdhb-1::mtLS::GFP*; *pRF4(rol-6)](hqls181)*; and N2.

### 2.2. RNAi Treatment

For RNAi experiments, standard NGM molten agar was supplemented with 1 mM isopropyl β-D-1-thiogalactopyranoside (IPTG) and 100 µL/mL carbenicillin. RNAi assays were conducted by culturing synchronized worms to the L4 stage on plates seeded with HT115 *E. coli* transformed with plasmids expressing control dsRNA (L4440) or gene-targeting dsRNA.

### 2.3. ^15^N Labeling of C. elegans

^15^N-labeled worms were prepared as described [28], and ^15^N-labeled *E. coli* MG1655 [28] was used as food, achieving a final ^15^N atomic enrichment of >95%.

### 2.4. Isolation of Mitochondria from C. elegans

Synchronized *C. elegans* worms were grown on NGM agar with *E. coli* OP50. Equal volumes of ^15^N-labeled and unlabeled *C. elegans* day-1 adults (5 mL packed worms each) were mixed and processed for fractionation. For mitochondrial isolation, homogenization of the worm mix was conducted in a pre-chilled 2 mL glass Dounce homogenizer (HEAD Biotechnology, Beijing, China) using 20 strokes with a pestle. The homogenate was then centrifuged at 750× *g* for 5 min at 4 °C to remove nuclei and debris. To further purify the homogenate from debris and bacteria, it was passed through a 5 μm PVDF syringe filter. The filtered homogenate was subsequently centrifuged at 9000× *g* for 10 min at 4 °C to further enrich the mitochondrial fraction. Next, the crude mitochondrial fraction was layered onto a 30% Percoll gradient and centrifuged at 95,000× *g* for 30 min using an Optima XE-100 Beckman centrifuge (SW55Ti rotor (Beckman, CA, USA). The pure mitochondria were frozen in liquid nitrogen and kept at −80 °C until use.

### 2.5. Mass Spectrometry Analysis (LC-MS/MS)

Proteins were precipitated from isolated mitochondrial fractions using methanol–chloroform extraction, as previously described. Each protein sample was re-suspended in 8 M urea, 100 mM Tris, pH 8.5, and the protein concentrations were determined using a BCA assay kit (ThermoFisher Scientific, Waltham, MA, USA). After dilution, reduction, and alkylation, proteins were digested with trypsin at 37 °C overnight in 2 M urea, 20 mM methylamine, 100 mM Tris, pH 8.5 before being quenched with 5% formic acid. Peptides were separated using an Easy-nLC1000 reversed-phase chromatography system (Thermo Fisher Scientific, MA, USA) that was directly interfaced with a Q-Exactive mass spectrometer (Thermo Fisher Scientific, MA, USA) for final protein identification.

### 2.6. Identification and Relative Quantitation of Proteins

Tandem mass spectra were searched against the *C. elegans* protein database (wormpep 235) using the ProLuCID search algorithm [29] to identify ^14^N- or ^15^N-labeled peptides. ProLuCID search results were filtered using DTASelect 2.0 [26] with a spectrum-level false discovery rate (FDR) threshold of ≤1%. Peptides with a Z score <4 or a precursor mass deviation >7 ppm were excluded. At least one peptide was required to identify a protein, and the protein-level FDR was set at ≤5%. Peptide ^14^N/^15^N ratios were calculated using the pQuant software [25] and normalized to the median ratio of the sample. To identify proteins with significant abundance changes between the *daf-2* mutant and WT, a Wilcoxon rank-sum test was performed on the ^14^N/^15^N ratio values of the constituent peptides. The resulting *p*-values were adjusted using the Benjamini–Hochberg procedure to control FDR.

### 2.7. Lifespan Assays

Lifespan was defined as the duration from the first day of adulthood until the worms were scored as dead. Worms that died of protruding/bursting vulva, bagging, or crawling off the agar were censored from the analysis. Lifespan assays of the *daf-2* mutant on RNAi bacteria were conducted at 25 °C. All the other lifespan assays were conducted at 20 °C. The log-rank method was applied to compare the survival curves.

### 2.8. Transmission Electron Microscopy (TEM)

TEM analysis of isolated mitochondria was performed as previously described [23]. Purified mitochondria were fixed in 2.5% glutaraldehyde in LB buffer at 41 °C for 2 h. After washing once with PBS, the pellet was post-fixed in 1% osmium tetroxide in PBS for 20 min at room temperature, then dehydrated in a gradient series of ethanol, carefully transferred to propylene oxide, and embedded in Epon-Araldite resin. Ultrathin sections (50–70 nm thick) were cut using a Leica EM UC6 Ultramicrotome (Leica, Milton Keynes, UK) with a diamond knife. The sections were picked up on formvar–carbon-coated copper grids, stained with saturated uranyl acetate followed by lead citrate, and examined with an electron microscope (Tecnai spirit) (FEI, OR, USA) at 120 kV.

### 2.9. Mitochondrial Ribosome Profiling

The isolated mitochondria were promptly washed three times and resuspended in 100 µL of ribosome buffer (110 mM KAc, 20 mM MgAc2, 10 mM HEPES pH 7.6, 100 mM KCl, 10 mM MgCl_2_, 0.1% NP-40, 2 mM DTT, and 40 U/mL RNasin). The mitochondria were then lysed with DEPC-treated 1 mL glass beads using a FastPrep^®^-24 instrument (MP Biomedicals, CA, USA) at 6.5 m/s for 20 s per pulse, repeated twice with 5 min cooling intervals. After lysing, supernatants were collected by centrifuging at 1200× *g* for 10 min at 4 °C. Protein concentrations were measured using a BCA protein assay. For ribosome profiling, protein samples were loaded onto preformed isokinetic sucrose gradients and centrifuged at 40,000 rpm for 2 h at 4 °C using a SW41 Ti rotor. The gradient was formed by overnight diffusion of four layers of sucrose (10%, 16.7%, 23.4%, and 30%) at 4 °C. The gradient fractions were then pumped into a UA-6 UV–vis detector (Teledyne ISCO, CA, USA) using a TrisTM peristaltic pump (Teledyne ISCO, CA, USA) with absorbance monitored at 250 nm.

### 2.10. ATP Measurement

ATP levels were assessed with the ATP Bioluminescence Assay Kit HS II (Roche, Basel, Switzerland) using a GloMax^®^-96 Microplate Luminometer (Promega, WI, USA) with quantifications adjusted for protein content. For sample preparation, 100 day-1 adult *C. elegans* were placed in 40 μL of the provided Cell Lysis Reagent from the kit, supplemented with a complete protease inhibitor cocktail (Roche, Basel, Switzerland). The lysates were processed with the FastPrep^®^-24 instrument (MP Biomedicals, CA, USA) as outlined earlier, subsequently boiled for 10 min, and centrifuged at 14,000 rpm for 5 min. The clear supernatants were immediately stored at −80 °C for future use.

### 2.11. Respiration Measurement

Oxygen consumption was measured in day-1 and day-9 adult *C. elegans* using an OROBOROS Oxygraph-2K. The worms were washed with M9 buffer and incubated for 30 min to clear intestinal bacteria. For the day-9 worms, 5-Fluoro-2′-deoxyuridine (FUDR) was used to inhibit progeny growth. Worm suspensions (50 µL packed worms in 500 µL M9 buffer) were placed in Oxygraph-2K chambers for respiration assays with FCCP treatments. Following oxygen consumption measurements, worms were lysed, and protein content was determined to normalize respiration rates.

### 2.12. Quantitation of Branched-Chain Amino Acids (BCAA)

For the BCAA assay, Buspinone (Sigma, St. Louis, MO, USA) was utilized as an internal standard. Stock solutions of L-Valine (Sigma), L-Isoleucine (Sigma), and L-Leucine (Sigma, MO, USA) were prepared in 50% ethanol. The day-1 adults of WT, *daf-2*, and the *daf-2*; *daf-16* double mutant were washed three times with M9 buffer, followed by two more washes with 50% methanol. The harvested worms were lysed in 200 μL of 50% methanol using a FastPrep^®®^-24 instrument (MP Biomedicals, CA, USA) as described above. The lysates were centrifuged at 4000× *g* for 5 min, the supernatants were collected for BCAA assays, and the pellets were dissolved in 4% SDS for the BCA protein assay.

### 2.13. mRNA-Seq

Total RNA was extracted from worms treated with RNAi using the Trizol reagent. mRNA libraries for mRNA-seq were prepared using the VAHTS Universal V8 RNA-seq Library Prep Kit (Vazyme, Nanjing, China) following the manufacturer’s protocol. The RNA-Seq reads data were then analyzed using a web-based RNA-Seq analysis platform of Westlake University.

### 2.14. Statistical Analysis

Full lifespan assays were evaluated using standard Kaplan–Meier log-rank survival tests, with the first day of adulthood of synchronized hermaphrodites defined as t = 0. Statistical significance was assessed using Student’s paired and unpaired *t*-tests for parametric data, or Wilcoxon rank-sum tests for non-parametric data; *p*-values ≤ 0.05 were considered statistically significant.

## 3. Results

### 3.1. daf-2 Mutant Maintains a Youthful Mitochondrial Morphology at an Old Age

**Figure 1:**
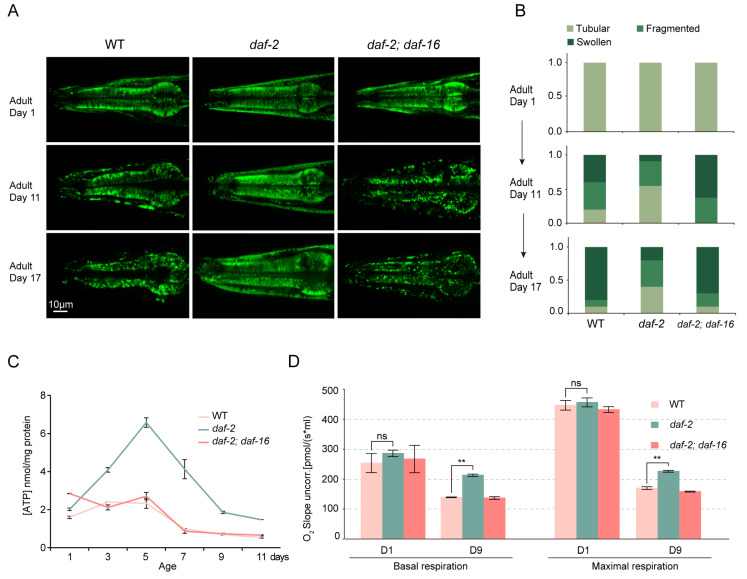
Mitochondrial activity in *daf-2* (*e1370*) mutants during aging. (**A**) Representative images of mitochondrial morphologies in the pharynx of WT, *daf-2*, *daf-2*; *daf-16* at days 1, 11, and 17. (**B**) Quantification of mitochondrial morphology in panel (**A**). (*n* = 10). (**C**) Age-dependent ATP levels in WT, *daf-2* mutants, and *daf-2*; *daf-16* mutants with age. (**D**) Respiration activity in WT, *daf-2*, and *daf-2*; *daf-16* worms at days 1 and 9 of adulthood. (** *p* value ≤ 0.01, ns indicates no statistical significance).

Aging is typically linked to a deterioration in mitochondrial function [30] and a progressive transition from an intricate, filamentous mitochondrial structure to a more granular form [31]. Furthermore, alterations in mitochondrial morphology can signal mitochondrial dysfunction [32]. To analyze the mitochondrial function in the long-lived *daf-2* mutant during aging, we first investigated the morphological changes in *daf-2* mitochondria over time using a mitochondrion-targeted GFP (mt-GFP) [33] as a marker for the pharynx (Figure 1A) and body wall muscle (Figure S1B). In the pharynx, we observed substantial mitochondrial disorganization in WT by day 11, whereas the *daf-2* mutant maintained a relatively normal arrangement up to day 17 (Figure 1B and Figure S1A). Likewise, in the body wall muscle, most WT mitochondria appeared swollen and fragmented by adult day 11, while *daf-2* mitochondria retained an elongated, well-organized network pattern until day 17 (Figure S1B). Together, these observations suggest that *daf-2* animals can maintain functional mitochondria over an extended aging period.

### 3.2. daf-2 Mutants Resist the Age-Related Decline in Mitochondrial Function

To assess whether the preservation of youthful mitochondrial morphology in *daf-2* mutants correlates with sustained functional integrity, we compared ATP content and oxygen consumption rates in young and aged *daf-2* mutants, WT animals, and *daf-2*; *daf-16* double mutants. As expected, ATP levels in *daf-2* mutants were significantly elevated from adult day 3 to 11 (Figure 1C), consistent with findings previously reported by Brys et al. [17]. Although basal respiration was similar among all groups on adult day 1, it declined more gradually in *daf-2* mutants on adult day 9 compared to WT and *daf-2*; *daf-16* double mutants. The mitochondrial uncoupler carbonilcyanide p-triflouromethoxyphenylhydrazone (FCCP) is commonly employed in mitochondrial function studies [34]. We observed that FCCP-induced respiration was similar among the WT, *daf-2* mutants, and *daf-2*; *daf-16* double mutants on adult day 1, but it was significantly higher in *daf-2* animals on adult day 9 (Figure 1D). Collectively, these findings suggest that mitochondrial function remains well-maintained even in aged *daf-2* mutants.

### 3.3. Quantitative Analysis of Mitochondrial Proteome in IIS-Regulated Long-Lived C. elegans

To explore the essential role of mitochondria in IIS-regulated longevity at the proteome level, we conducted a quantitative proteomic analysis following mitochondrial purification [35,36]. Using ^15^N labeling of mitochondria as a reference, we accurately evaluated changes in protein levels across multiple samples within a single experiment [37]. This approach enabled systematic investigation of changes in mitochondrial protein abundance in IIS-deficient *C. elegans* (Figure S2A). To confirm the purity of mitochondria isolated through our workflow, we employed two distinct validation methods. First, transmission electron microscopy (TEM) was used to visually confirm successful mitochondrial enrichment (Figure S2B). Subsequently, mitochondrial purity was assessed through immunoblotting analyses. Results indicated substantial mitochondrial protein (PDHE1-a) enrichment in the mitochondrial fraction (MF) following multiple fractionation steps, with minimal residual plasma membrane proteins (UAP56) remaining (Figure S2C).

**Figure 2:**
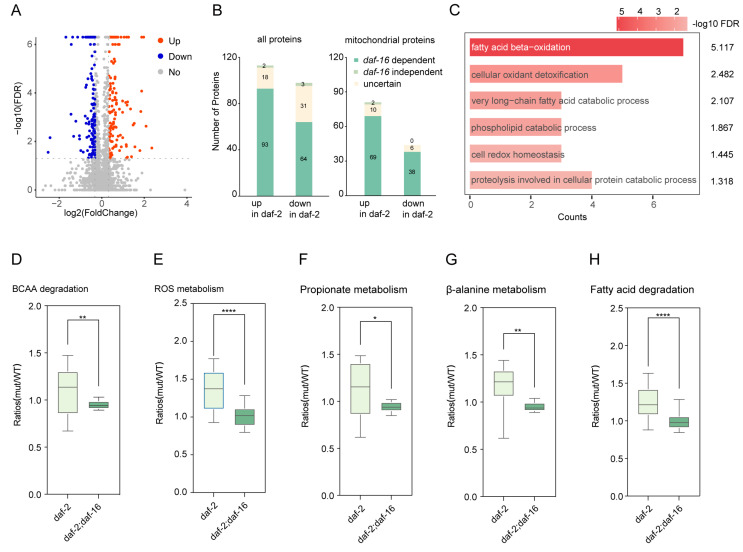
Quantitative proteomics of mitochondria from long-lived *daf-2* (*e1370*) mutants. (**A**) Volcano plot of the significantly dysregulated proteins. (|log2 fold-change| > 1.25, FDR < 0.05). *n* = 3 biological replicates. (**B**) Number of proteins with differential abundance in *daf-2* (*e1370*) mutants relative to wild-type (**left**) and differentially expressed mitochondrial proteins (**right**). (**C**) Gene ontology (GO) enrichment analysis of upregulated proteins in (**A**) (FDR < 0.05). (**D**–**H**) Boxplots showing quantified proteins in upregulated metabolic pathways including BCAA (branched-chain amino acids), ROS (reactive oxygen species), propionate, β-alanine, and fatty acid in *daf-2* mutants relative to WT. (* *p* value ≤ 0.05, ** *p* value ≤ 0.01, **** *p* value ≤ 0.0001).

To enhance the precision of quantification, we conducted three independent biological replicates. More than 1600 proteins were identified using Prolucid software with a false discovery rate (FDR) below 1% at the protein level, with each biological replicate sample containing at least one unique peptide. Of these proteins, over 1000 were consistently observed across all three biological replicates (Figure S2D), indicating a high reproducibility across independent experiments. In total, we identified 2425 proteins in a comparison of the *daf-2* mutant and WT (Table S1). Among the 2425 quantified proteins, 257 were identified as differentially expressed, with 134 proteins showing upregulation (FC > 1.25, FDR < 0.05, Table S2) and 123 proteins showing downregulation (FC < 0.8, FDR < 0.05, Table S2). The majority of these changes depended on the activity of the FOXO transcription factor DAF-16 (Figure 2B), suggesting that DAF-16 acts as a key regulator in modulating mitochondrial function within the IIS pathway. We compared our data set with published transcriptomics (Murphy et al. 2003) and proteomics (Dong et al. 2007, Detuydt et al. 2014) of the IIS pathway [37,38,39] (Figure S2G). Among these, only 34 proteins overlapped. Therefore, we effectively identified the proteins that were altered in the *daf-2* mutant compared to the wild type.

### 3.4. Differentially Expressed Proteins in ROS and Mitochondrial Intermediary Metabolisms of daf-2 Mutants

To further investigate the biological implications of mitochondrial protein changes in *daf-2* mutants, we first performed gene ontology (GO) analysis on the upregulated proteins (Figure 2C). Results indicated enrichment of biological processes in upregulated proteins, including fatty acid beta-oxidation (GO:0006635), peroxisome organization (GO:0007031), and cellular oxidant detoxification (GO:0098869). Furthermore, KEGG pathway analysis highlighted five fundamental enriched metabolic pathways: branched-chain amino acid (BCAA) degradation, peroxisome (linked to ROS metabolism), propionate catabolism, beta-alanine metabolism, and fatty acid (FA) degradation [39,40]. Notably, the inactivation of DAF-16 completely abolished the activation of these pathways in *daf-2* mutants (Figure 2D–H and Figure S2F) (Tables S6 and S7), suggesting a potential link between these metabolic pathways and the extended lifespan observed in *daf-2* mutants. Together, these findings reveal that mitochondria in *daf-2* mutants display distinct metabolic changes compared to those in WT and *daf-2*; *daf-16* double mutants.

**Figure 3:**
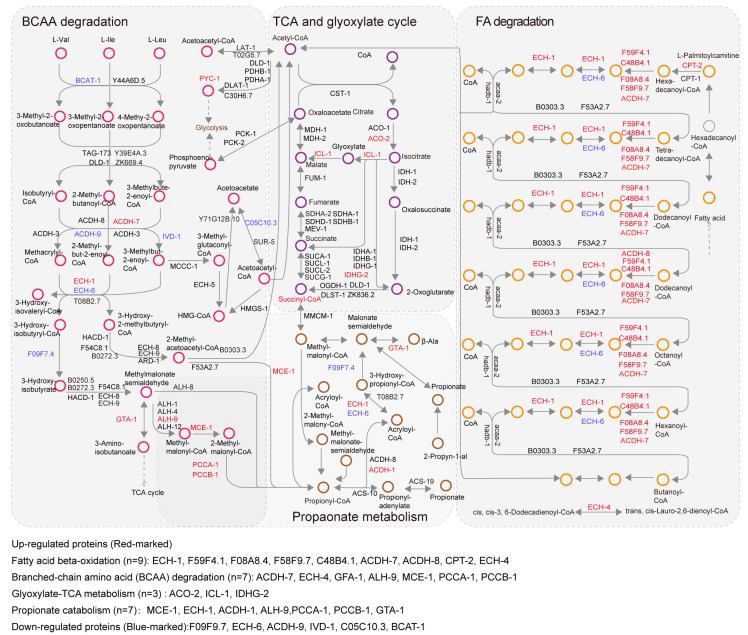
Map of interaction network among fatty acid degradation, glyoxylate cycle, propionate metabolism, TCA cycle, and BCAA catabolism. Proteins marked in red or blue indicate up- or downregulation in *daf-2* mutant worms, respectively.

To systematically characterize these mitochondrial intermediary metabolisms, we constructed an interaction network of metabolic pathways based on KEGG pathway annotations. Three metabolites, acetyl-CoA, propionyl-CoA, and succinyl-CoA, emerged as central nodes, linking these processes into an interconnected metabolic module (Figure 3). Surprisingly, 50 quantified proteins were involved in this metabolic network, with 19 upregulated and only 6 downregulated. Notably, upregulated proteins were primarily associated with fatty acid beta-oxidation (*n* = 9), branched-chain amino acid (BCAA) degradation (*n* = 7), glyoxylate-TCA metabolism (*n* = 3), and propionate catabolism (*n* = 7). Upregulation of these metabolic pathways suggests a coordinated regulatory pattern likely governed by the activities of DAF-2 and DAF-16 (Figure 2D–H). These findings suggest that synchronized alterations in these metabolic processes may play a role in lifespan extension in the *daf-2* (*e1370*) mutant.

### 3.5. Enhanced BCAA Metabolism Promotes the Lifespan Extension of C. elegans

To investigate the role of upregulated mitochondrial BCAA metabolism (Figure 2D) in lifespan extension, we performed lifespan assays with ALH-9 (aldehyde dehydrogenase involved in BCAA metabolism) in this pathway. As expected, RNAi-mediated silencing of ALH-9 shortened the lifespan of *daf-2* mutants (Figure S3A). Previous studies found that BCAA supplementation extended the chronological lifespan of yeast and promoted survival in mice [41]. Inspired by these findings, we examined whether long-lived *daf-2* mutants exhibit elevated BCAA levels. We found that BCAA levels increased approximately two-fold in *daf-2* mutants compared to both WT and *daf-2*; *daf-16* worms (Figure S2E). This finding is consistent with the observed downregulation of BCAT-1 in impaired IIS *daf-2* mutants, also supporting the previous studies’ findings that BCAA supplementation increases lifespan in *C. elegans* [42,43]. These findings suggest that higher BCAA levels likely benefit the *daf-2*-induced longevity. Overall, enhanced BCAA metabolism promotes longevity in *daf-2* mutants.

### 3.6. Upregulation of Propionate, FA, and β-Alanine Metabolisms Contributes to the Longevity of daf-2 Mutants

**Figure 4:**
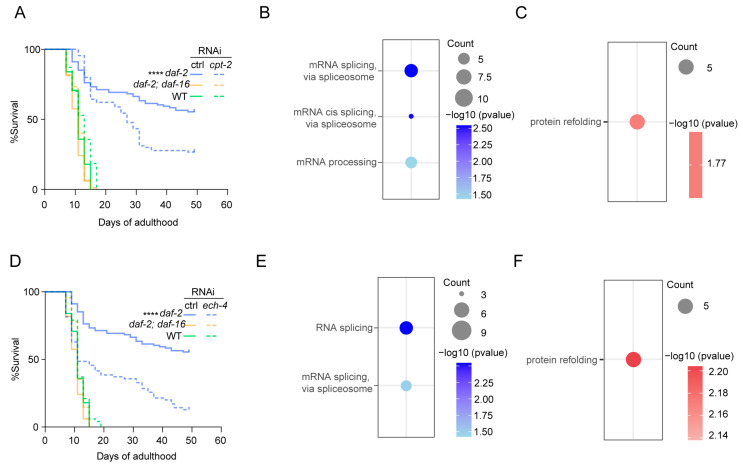
RNAi inactivation of metabolic genes shortened the lifespan of *daf-2* (*e1370*) mutants by upregulating the protein refolding process via mRNA-seq analysis. (**A**,**D**) Lifespan analysis of RNAi-treated WT, *daf-2*, and *daf-2*; *daf-16* worms. (**A**) *cpt-2* RNAi. (**D**) *ech-4* RNAi. Lifespan data are plotted as Kaplan–Meier survival curves, and the *p*-value was calculated using Mantel–Cox log-ranking (**** *p*-value ≤ 0.0001, *n* = 100 for each condition). (**B**,**E**) Gene enrichment analysis for downregulated genes in RNAi-treated *daf-2* worms. (**B**) *cpt-2* RNAi. (**E**) *ech-4* RNAi. (*p*-value < 0.05). (**C**,**F**) Gene enrichment analysis for upregulated genes of *daf-2* worms treated with RNAi. (**C**) *cpt-2* RNAi treatment. (**F**) *ech-4* RNAi. (*p*-value < 0.05).

To determine whether enhanced mitochondrial intermediate metabolism supports longevity, we performed lifespan assays using RNAi to knock down candidates whose abundance was elevated in *daf-2* mutants. ECH-1, an enoyl-CoA hydratase involved in FA, BCAA, and propionate metabolism, was significantly increased in *daf-2* mutants. RNAi-mediated knockdown of ECH-1 significantly reduced the *daf-2* mutant lifespan in a *daf-16*-dependent manner (Figure S3B). RNAi-mediated inhibition of enoyl-CoA isomerase (ECH-4) and carnitine palmitoyltransferase (CPT-2), both involved in FA degradation, also considerably reduced *daf-2* mutant lifespan (Figure 4A,D). Additionally, GTA-1 deficiency (an ortholog of human GABA transaminase involved in malonate semialdehyde processing) shortened the *daf-2* mutant lifespan but did not affect the WT lifespan (Figure S3C). Lastly, ICL-1, a key enzyme in the glyoxylate cycle, exhibited a two-fold increase in abundance in *daf-2* mutants compared to WT or *daf-2*; *daf-16* nematodes, consistent with Murphy’s report in 2003 [38]. Previous observations also indicate that *icl-1* mutants live shorter than WT [33,38]. Collectively, these findings suggest that coordinated mitochondrial metabolic modulation underlies *daf-2* mutant longevity.

To further understand the transcriptome changes corresponding to metabolic alteration in *daf-2* mutants, we performed mRNA-Seq analyses of *C. elegans*, with a particular focus on FA metabolism as an example. For the FA degradation process, both *cpt-2* and *ech-4* RNAi knockdown were conducted. We identified 163 upregulated genes in the *cpt-2* RNAi-treated animals and 209 in the *ech-4* RNAi-treated animals (Figure S3D,E; Tables S3 and S4). Gene expression changes in both RNAi-treated animal groups displayed similar transcriptional signatures—protein refolding enrichment (GO:0042026) (Figure 4C,F), implying the involvement of accumulated unfolded or abnormally folded proteins in the FA degradation process-mediated aging, as reported in previous studies [44,45]. In addition, for the 164 and 108 genes that showed a decreased expression level in the respective *cpt-2* and *ech-4* RNAi groups, the transcripts were primarily enriched in the RNA splicing process (GO:0000398, GO:0045292, GO:0003880) (Figure 4B,E). Consistently, previous reports have revealed that overexpression of SFA-1 (Splicing Factor 1) can extend the lifespan of *C. elegans* [46]. These data suggest that upregulated FA degradation in long-lived *daf-2* mutants partially promotes longevity by affecting the protein folding process and RNA splicing activity. Collectively, our data suggest that the downregulated activity of propionate, FA, and β-alanine metabolism likely contributes to *daf-2* longevity, highlighting the role of these mitochondrial intermediate metabolisms in lifespan extension.

### 3.7. Fine-Tuning in Mitochondrial Translation Contributes to IIS-Mediated Longevity

**Figure 5:**
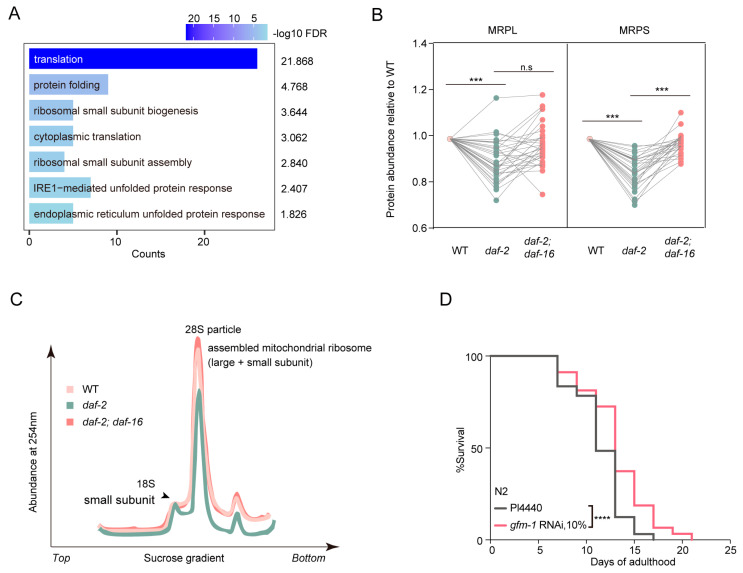
Reduced mitochondrial translation machinery in *daf-2* (*e1370*) mutants contributes to lifespan extension. (**A**) Gene ontology (GO) enrichment analysis of downregulated proteins in *daf-2* (*e1370*) mitochondria compared to WT (FDR < 0.05). (**B**) Abundance of mitochondrial ribosome proteins in WT, *daf-2*, and *daf-2*; *daf-16* mutants. MRPL: mitochondrial ribosomal proteins from large subunits; MRPS: mitochondrial ribosomal proteins from small subunits. (*** *p*-value ≤ 0.0001, ns indicates no statistical significance) (**C**) Representative traces of mitochondrial ribosome profile in young adulthood of WT (pink), *daf-2* (green), and *daf-2*; *daf-16* (orange) worms. (**D**) Lifespan analysis of diluted *gfm-1* RNAi-treated worms compared with control. (**** *p*-value ≤ 0.0001, *n* = 100 for each condition).

Beyond the mitochondrial metabolic changes, gene ontology (GO) analysis of downregulated mitochondrial proteins in *daf-2* mutants pointed to the “translation” process (GO:0006412) (Figure 5A). Cytoplasmic translation activity is known to be reduced in *daf-2* mutants [47], yet mitochondrial translation activity has not been previously explored. Here, many mitochondrial ribosomal proteins showed significantly reduced levels (*p* < 0.001) in *daf-2* mutants compared to WT counterparts (Figure 5B). To confirm this observation, we conducted mitochondrial ribosome profiling in WT, *daf-2*, and *daf-2*; *daf-16* double mutants. Ribosomes were separated using sucrose gradient centrifugation, and bound ribosomal RNAs (rRNAs) were quantified via absorbance at 254 nm. Compared to WT *C. elegans*, *daf-2* mutants exhibited a notable decrease in rRNAs at the 28S peak (assembly of small and large ribosomal subunits) (Figure 5C). This finding aligns with the observed reduction in ribosomal protein abundance, suggesting that the *daf-2(e1370)* mutation might lead to decreased mitochondrial translation. This reduction is completely suppressed by *daf-16* mutation, implying that the reduced translation machinery may be involved in impaired insulin-signal-mediated longevity. Together, these observations might suggest a reduction in de novo protein synthesis in *daf-2* mitochondria.

To investigate the role of reduced mitochondrial translation in IIS-regulated longevity, we inhibited the mitochondrial translation elongation factor GFM-1 in WT, which is downregulated in *daf-2* mutants, and performed lifespan assays. Given the mild reduction in mitochondrial translation machinery in *daf-2* mutants, we used diluted RNAi (10%) bacteria to mildly knock down targets. We observed that inhibition of GFM-1 (FC = 0.82) significantly extended *C. elegans* lifespan, whereas undiluted RNAi-mediated suppression shortened lifespan (Figure 5D and Figure S4A,B). This result confirms that mildly tuning down mitochondrial translation activity might contribute to Insulin/IGF-1-mediated longevity, suggesting a role for mitochondrial translation activity in lifespan extension in *C. elegans*.

### 3.8. Suppression of Mitochondrial Translation Decreases the Activity of the Lipid Metabolic Process and Cytoplasm Fatty Acid Elongation

**Figure 6:**
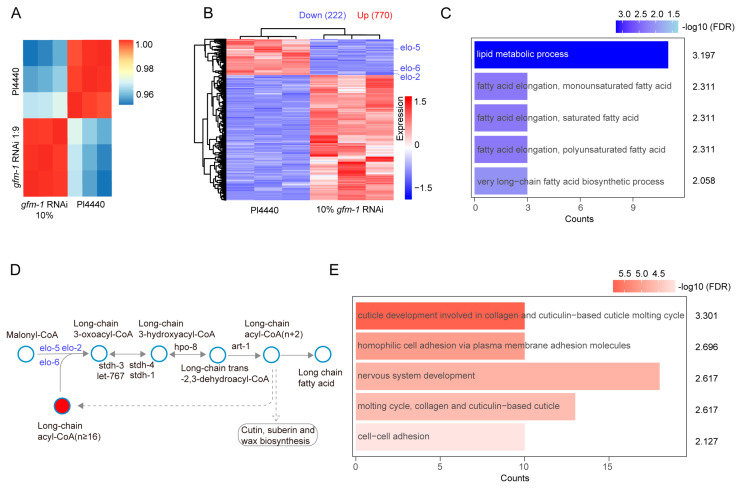
mRNA sequencing analysis of WT worms following a slight reduction in mitochondrial translation activity. (**A**) Principal component analysis (PCA) of L4440 and diluted *gfm-1* RNAi-treated samples. (**B**) Heatmap of the significantly dysregulated mRNAs (|log2 fold-change| > 1, *p*-value < 0.05). (**C**) Gene enrichment analysis for downregulated genes in the *gfm-1* RNAi group compared to L4440. (**D**) Schematic diagram of fatty acid elongation in the cytoplasm. (**E**) Gene enrichment analysis for upregulated genes in the *gfm-1* RNAi group compared to L4440.

To further investigate the potential reasons why mitochondrial translation activity affects lifespan, we conducted whole transcriptome analysis of WT *C. elegans* on adult day 1, fed with control RNAi (L4440) and the diluted *gfm-1* RNAi bacteria, respectively. Gene expression measured by mRNA-seq showed a high correlation among three independent biological repeats for control RNAi or *gfm-1* RNAi treatment (Figure 6A). The data revealed 770 upregulated genes and 222 downregulated genes following mild knockdown of *gfm-1* (Figure 6B, Table S5). Gene enrichment analysis indicated that downregulated genes were most enriched in lipid metabolism and fatty acid elongation (GO:0006629, GO:0034625) (Figure 6C). Notably, among those downregulated genes, we identified three enzymes of the ELOVL (elongation of very long-chain fatty acids) family, including ELO-2, ELO-5, and ELO-6 in the cytoplasm (Figure 6D,E). Overall, our results suggest that reduced mitochondrial translation promotes lifespan extension in *C. elegans,* likely by modulating lipid metabolic processes and cytoplasmic fatty acid chain elongation.

Previous studies have shown that the level of β PIX-GIT (focal adhesion-localized β PAK-interacting exchange factor) decreases significantly with aging [48]. This decline leads to impaired turnover of focal adhesions and dysregulated integrin signaling, ultimately resulting in increased production of reactive oxygen species (ROS) and subsequent entry into senescence. Here, our data show that 770 upregulated transcripts in *gfm-1* RNAi-treated worms are enriched in cell adhesion, suggesting a positive association between cell adhesion and longevity (Figure 6E, GO:0007156, GO:0098609). Together, these findings reveal that reduced mitochondrial translation might regulate downstream processes of lipid metabolism and cell adhesion pathways, which are likely associated with an extended lifespan due to *gfm-1* RNAi.

## 4. Discussion

In this research, we identified the mitochondrial proteome and profiled mitochondrial metabolic tuning in *daf-2* mutants, a key gene encoding an insulin receptor family member. The mitochondrial metabolic modulations likely contribute to the lifespan extension of *daf-2* mutants. *daf-2* mutation-mediated mitochondrial metabolic changes include branched-chain amino acids (BCAAs), reactive oxygen species (ROS), β-alanine, propionate, and fatty acids (FAs). RNAi knockdown of metabolic enzymes involved in these pathways (CPT-2, ECH-4, GTA-1) decreases the lifespan of *daf-2* mutants. Lastly, compared to WT *C. elegans*, mitochondrial ribosome quantity might be reduced in *daf-2* mutants, contributing to lifespan extension likely through modulation of lipid metabolism. Our research revealed a proteome-wide alteration of mitochondrial proteins in long-lived *daf-2* animals, revealing the role of mitochondrial metabolic tuning in lifespan extension.

Mitochondrial functional alterations are well-documented to be closely linked with progressive functional decline during aging [49,50]. Previous studies of the *C. elegans* IIS pathway revealed altered expression of numerous metabolic pathway genes and proteins through microarray and proteomics analyses [37,38]. However, limited mitochondrial protein coverage in whole *C. elegans* proteomics posed challenges in identifying a comprehensive set of mitochondrial proteins in proteomic studies. Given the pivotal role of mitochondria in regulating metabolism and aging [33,51], we purified mitochondria and performed proteomic analysis to investigate the abundance changes of mitochondrial proteins specifically. Our findings suggest that IIS-modulated longevity is uniquely characterized by coordinated upregulation of mitochondrial metabolic pathways, including fatty acid β-oxidation, branched-chain amino acid degradation, the tricarboxylic acid (TCA) cycle, glyoxylate metabolism, and propionate catabolism (Figure 2). Furthermore, these pathways are closely interconnected through the action of three well-established multifunctional intermediates: succinyl-CoA, propionyl-CoA, and acetyl-CoA [52] (Figure 3). These alterations in metabolic flux suggest a reorganization of the metabolic network in *daf-2* mitochondria. Thus, it is plausible that *daf-2* mutant mitochondria undergo a series of metabolic adjustments that support longevity assurance. In addition, mitochondrial biogenesis may play a crucial role in the metabolic changes observed in *daf-2* mutants. Previous reports have suggested that increased mitochondrial mass supports enhanced metabolic activities such as fatty acid oxidation and the TCA cycle [53,54]. Therefore, future studies quantifying mitochondrial biogenesis—such as measuring mitochondrial content or specific markers—will help further understand how these metabolic pathways are integrated at the mitochondrial level.

We propose that fine-tuning mitochondrial metabolism in *daf-2* mutants may enhance the conversion of lipids and BCAAs to carbohydrates. First, it has been reported that *daf-2* mutants are significantly characterized by elevated lipid levels [55]. Given our finding that most enzymes in fatty acid β-oxidation are upregulated, we speculate that more acetyl-CoA generated from fatty acid β-oxidation is funneled into *daf-2* mitochondrial carbohydrate metabolism. Additionally, catabolism of branch-chain amino acids (BCAAs), including Val, Leu, and Ile, is an important pathway in mitochondria. Upon degradation, BCAAs are metabolized into acetyl-CoA and succinyl-CoA, which serve as essential intermediates in the TCA and glyoxylate cycles. The elevated BCAA levels and modest upregulation of the BCAA pathway (Figure 2D) suggest that more intermediates from BCAA metabolism might enter the TCA and glyoxylate cycles. The observed increase in propionate levels in *daf-2* mutants further suggests BCAA catabolism activation, as propionate is a direct byproduct of valine and isoleucine degradation [56]. Furthermore, propionate is recycled into the TCA cycle as succinyl-CoA via propionate metabolism (Figure 3). In summary, our findings identified upregulation of this metabolic pathway and suggested an increased influx of succinyl-CoA into the TCA cycle. Thus, *daf-2* mitochondria may sustain energy supply by utilizing carbohydrates derived from lipids and BCAAs.

BCAA degradation also intersects with major longevity-regulating pathways, including insulin/IGF-1 signaling (IIS), mTOR, and AMPK pathways, thereby influencing energy metabolism and lifespan extension. Among BCAAs, leucine is a potent activator of mTORC1, and excessive mTORC1 activation has been implicated in aging [57,58]. Upregulation of BCAA catabolism may attenuate mTORC1 signaling, thereby promoting autophagy and enhancing mitochondrial quality control, both of which are well-established contributors to longevity [11]. Furthermore, BCAA degradation yields acetyl-CoA and succinyl-CoA, which fuel the TCA cycle and modulate IIS signaling by adjusting cellular nutrient availability, thereby influencing lifespan regulation. Additionally, BCAA catabolism may indirectly activate AMPK, facilitating metabolic reprogramming, improving mitochondrial function, and maintaining cellular energy homeostasis [59]. Collectively, BCAA metabolism may serve as a central metabolic node that integrates IIS, mTOR, and AMPK signaling to coordinate nutrient sensing, autophagy, and mitochondrial maintenance, ultimately promoting longevity. Future studies leveraging multi-omics approaches will be crucial for systematically delineating the mechanistic role of BCAA metabolism in lifespan regulation and advancing our understanding of the aging process.

The conservation of mitochondrial function and its involvement in aging across species suggests broader relevance. For instance, key metabolic pathways identified in this study, such as fatty acid β-oxidation and BCAA catabolism, have also been implicated in aging and longevity in mammals [58,60]. Elevated lipid levels and altered lipid metabolism, as observed in *daf-2* mutants, are consistent with findings in murine models where mitochondrial metabolic reprogramming supports longevity [2,61]. Moreover, mitochondrial translation and ribosomal activity, shown here to impact lifespan in *daf-2* mutants, align with observations in mice, where partial inhibition of mitochondrial translation enhances healthspan and lifespan [62,63]. These parallels emphasize the evolutionary conservation of mitochondrial mechanisms in regulating aging.

Mitochondrial dysfunction, a hallmark of aging, is associated with reduced energy production, elevated reactive oxygen species (ROS) levels, and impaired proteostasis [64]. Protein misfolding and aggregation within mitochondria compromise mitochondrial function and contribute to the aging process [65]. In line with this, we observed that the protein refolding process was upregulated in *daf-2* mutants when subjected to knockdown of *ech-4* and *gta-1*, which shortened the lifespan of *daf-2* mutants. Additionally, prior studies have shown that various ribosomal proteins (such as RPS-15 and RPS-22) and translation factors (e.g., eIF2β and eIF4G) play roles in *C. elegans* lifespan extension [47,66]. Notably, Stout et al. also demonstrated a reduction in numerous cytoplasmic ribosomal proteins in long-lived *daf-2* mutants, emphasizing its significance for lifespan extension [47]. In the study by Narayan et al. (2016), the age-related proteins they identified pointed to changes in lipid transport and metabolic processes [21]. Through our mitochondrial quantitative proteomics study, we further discovered the significance of mitochondrial fatty acid catabolism in aging. Additionally, in the study by Walther et al. (2015) [20], it was found that mitochondrial protein levels decrease with age. Notably, the ratio of mitochondrial ribosomal proteins to cytosolic ribosomal proteins shows a smaller loss. This observation raises the question of whether there is a connection between mitochondrial translation activity and cytosolic translation during aging, which warrants further investigation in future research. Notably, our findings also suggest that ELO-2, ELO-5, and ELO-6 likely play a role in lifespan extension. Members of the ELOVL (elongation of very long-chain fatty acids) family are crucial for elongating fatty acid chains. Currently, nine elo genes have been identified in the *C. elegans* genome, and their metabolic functions are partially characterized [67]. *elo-2*, *elo-5*, and *elo-6* may influence lifespan by regulating this fatty acid elongation process [68]. Studies on the role of ELOVL enzymes in fatty acid metabolism and lifespan extension will help elucidate how lipid metabolism influences aging and provide a theoretical foundation for developing aging intervention strategies based on metabolic regulation.

## 5. Conclusions

In conclusion, our study quantified the mitochondrial proteome of IIS-deficient *C. elegans*, providing insights into the regulatory role of mitochondria in aging. We found that *daf-2* mitochondria exhibit coordinated changes in intermediary metabolism, and a modest reduction in translation machinery compared to WT during aging. These characteristics of *daf-2* mitochondria contribute to longevity assurance to some extent. Overall, these findings underscore the role of mitochondria in organismal aging and IIS-deficient-regulated longevity. Given the conservation of these mitochondrial proteins, studies of potential mitochondrial adaptations in mammals may provide new insights into aging processes and age-related diseases.

## Data Availability

The original contributions presented in the study are included in the article/Supplementary Materials. mRNA seq data are available in the NCBI GEO database: accession number GSE274456.

## Supplementary Materials

The following supporting information can be downloaded at https://www.mdpi.com/article/10.3390/biology14030279/s1. Figure S1. Age-dependent mitochondrial function decline was attenuated by mutation of *daf-2*. (A) Representative images of tubular, fragmented, and swollen mitochondrial morphology in the pharynx. (B) Representative images of mitochondrial morphologies in body wall muscle of WT, *daf-2*, *daf-2*; *daf-16* at days 1, 11, and 17. Figure S2. Quantitative mass spectrometry analysis of mitochondrial in WT, daf-2 and daf-2; daf-16 worms. (A) Workflow of mitochondria quantitative proteomic analysis. Int Std: internal standard. (B) Transmission electron microscopy (TEM) images of isolated mitochondria. (C) Western blotting verifying the purity of isolated mitochondria. MF: Mitochondrial Fraction; PNS: post-nucleus supernatant fraction; PMS: post-mitochondria supernatant; CM: Crude Mitochondria. (D) Overlap of identified proteins across biological replicates of mitochondrial samples from WT, *daf-2* mutant, *daf-2*; *daf-16* mutant. (E) The abundance of Valine, Isoleucine, and Leucine in WT, *daf-2* mutant, and *daf-2*; *daf-16* mutant showed by absolute quantification. (** *p* value ≤ 0.01, *** *p* value ≤ 0.001). (F) Statistical analysis of quantified proteins of wildtype, *daf-2* mutant, and *daf-2*; *daf-16* mutant in five metabolic pathways. (G) The Venn Diagram shows the comparison of different published data sets with our results. Figure S3. Lifespan and mRNA-seq analysis of *daf-2* (e1370) worms treated by RNAi of three metabolic enzymes. (A-C) Lifespan analysis of RNAi-treated WT, *daf-2*, and *daf-2*; *daf-16* worms. (A) *alh-9* RNAi. (D) *ech-1* RNAi. (C) *gta-1* RNAi. (D-E) Volcano plot of differentially expressed genes (DEGs) of WT, *daf-2*, *daf-2*; *daf-16* mutants treated with RNAi. (D) *cpt-2* RNAi treatment. (E) *ech-4* treatment. (|Fold-change| > 1.25, FDR < 0.05). (F) BCAA levels of day 1 adult *daf-2* mutant treated with *alh-9* RNAi. Figure S4. Lifespan analysis following RNAi knockdown of GFM-1 in WT, *daf-2*, and *daf-2*; *daf-16* mutants with (B) or without (A) RNAi dilution. Table S1. Proteins Identified by Quantitative Proteomic Analysis. Table S2. Differentially Expressed Proteins in *daf-2* mutant. Table S3. Differentially Expressed Genes in Response to *cpt-2* RNAi. Table S4. Differentially Expressed Genes in Response to *ech-4* RNAi. Table S5. Differentially Expressed Genes in Response to *gfm-1* RNAi. Table S6 Annotated proteins in five KEGG enriched metabolic pathways. Table S7 Quantified ratios of enriched metabolic pathways in wildtype, *daf-2* mutant, and *daf-2*; *daf-16* mutant.

## References

[1] de Magalhaes J.P. (2023). Ageing as a software design flaw. Genome Biol..

[2] Lopez-Otin C., Blasco M.A., Partridge L., Serrano M., Kroemer G. (2023). Hallmarks of aging: An expanding universe. Cell.

[3] Mammucari C., Rizzuto R. (2010). Signaling pathways in mitochondrial dysfunction and aging. Mech. Ageing Dev..

[4] Annesley S.J., Fisher P.R. (2019). Mitochondria in Health and Disease. Cells.

[5] Harman D. (1956). Aging: A theory based on free radical and radiation chemistry. J. Gerontol..

[6] Linford N.J., Schriner S.E., Rabinovitch P.S. (2006). Oxidative damage and aging: Spotlight on mitochondria. Cancer Res..

[7] Dai D.F., Santana L.F., Vermulst M., Tomazela D.M., Emond M.J., MacCoss M.J., Gollahon K., Martin G.M., Loeb L.A., Ladiges W.C. (2009). Overexpression of catalase targeted to mitochondria attenuates murine cardiac aging. Circulation.

[8] Bahadorani S., Hur J.H., Lo T., Vu K., Walker D.W. (2010). Perturbation of mitochondrial complex V alters the response to dietary restriction in Drosophila. Aging Cell.

[9] Bonafe M., Barbieri M., Marchegiani F., Olivieri F., Ragno E., Giampieri C., Mugianesi E., Centurelli M., Franceschi C., Paolisso G. (2003). Polymorphic variants of insulin-like growth factor I (IGF-I) receptor and phosphoinositide 3-kinase genes affect IGF-I plasma levels and human longevity: Cues for an evolutionarily conserved mechanism of life span control. J. Clin. Endocrinol. Metab..

[10] Holzenberger M., Dupont J., Ducos B., Leneuve P., Geloen A., Even P.C., Cervera P., Le Bouc Y. (2003). IGF-1 receptor regulates lifespan and resistance to oxidative stress in mice. Nature.

[11] Tatar M., Bartke A., Antebi A. (2003). The endocrine regulation of aging by insulin-like signals. Science.

[12] Post S., Liao S., Yamamoto R., Veenstra J.A., Nassel D.R., Tatar M. (2019). Drosophila insulin-like peptide dilp1 increases lifespan and glucagon-like Akh expression epistatic to dilp2. Aging Cell.

[13] Kenyon C., Chang J., Gensch E., Rudner A., Tabtiang R. (1993). A *C. elegans* mutant that lives twice as long as wild type. Nature.

[14] Tissenbaum H.A., Ruvkun G. (1998). An insulin-like signaling pathway affects both longevity and reproduction in Caenorhabditis elegans. Genetics.

[15] Lin K., Dorman J.B., Rodan A., Kenyon C. (1997). daf-16: An HNF-3/forkhead family member that can function to double the life-span of Caenorhabditis elegans. Science.

[16] Ogg S., Paradis S., Gottlieb S., Patterson G.I., Lee L., Tissenbaum H.A., Ruvkun G. (1997). The Fork head transcription factor DAF-16 transduces insulin-like metabolic and longevity signals in *C. elegans*. Nature.

[17] Brys K., Castelein N., Matthijssens F., Vanfleteren J.R., Braeckman B.P. (2010). Disruption of insulin signalling preserves bioenergetic competence of mitochondria in ageing Caenorhabditis elegans. BMC Biol..

[18] Roy C., Molin L., Alcolei A., Solyga M., Bonneau B., Vachon C., Bessereau J.L., Solari F. (2022). DAF-2/insulin IGF-1 receptor regulates motility during aging by integrating opposite signaling from muscle and neuronal tissues. Aging Cell.

[19] Traa A., Gonzalez A.A.T., Van Raamsdonk J.M. (2024). Developmental disruption of the mitochondrial fission gene drp-1 extends the longevity of daf-2 insulin/IGF-1 receptor mutant. Geroscience.

[20] Walther D.M., Kasturi P., Zheng M., Pinkert S., Vecchi G., Ciryam P., Morimoto R.I., Dobson C.M., Vendruscolo M., Mann M. (2015). Widespread Proteome Remodeling and Aggregation in Aging, *C. elegans*. Cell.

[21] Narayan V., Ly T., Pourkarimi E., Murillo A.B., Gartner A., Lamond A.I., Kenyon C. (2016). Deep Proteome Analysis Identifies Age-Related Processes in *C. elegans*. Cell Syst..

[22] Chen X., Wei S., Ji Y., Guo X., Yang F. (2015). Quantitative proteomics using SILAC: Principles, applications, and developments. Proteomics.

[23] Souza G., Guest P.C., Martins-de-Souza D. (2017). LC-MS(E), Multiplex MS/MS, Ion Mobility, and Label-Free Quantitation in Clinical Proteomics. Methods Mol. Biol..

[24] Maccarrone G., Chen A., Filiou M.D. (2017). Using (15)N-Metabolic Labeling for Quantitative Proteomic Analyses. Methods Mol. Biol..

[25] Liu C., Song C.Q., Yuan Z.F., Fu Y., Chi H., Wang L.H., Fan S.B., Zhang K., Zeng W.F., He S.M. (2014). pQuant improves quantitation by keeping out interfering signals and evaluating the accuracy of calculated ratios. Anal. Chem..

[26] Tabb D.L., McDonald W.H., Yates J.R. (2002). DTASelect and Contrast: Tools for assembling and comparing protein identifications from shotgun proteomics. J. Proteome Res..

[27] Byerly L., Cassada R.C., Russell R.L. (1976). The life cycle of the nematode *Caenorhabditis elegans*. I. Wild-type growth and reproduction. Dev. Biol..

[28] Khan Z., Amini S., Bloom J.S., Ruse C., Caudy A.A., Kruglyak L., Singh M., Perlman D.H., Tavazoie S. (2011). Accurate proteome-wide protein quantification from high-resolution 15N mass spectra. Genome Biol..

[29] Xu T., Park S.K., Venable J.D., Wohlschlegel J.A., Diedrich J.K., Cociorva D., Lu B., Liao L., Hewel J., Han X. (2015). ProLuCID: An improved SEQUEST-like algorithm with enhanced sensitivity and specificity. J. Proteom..

[30] de Boer A., Ter Horst G.J., Lorist M.M. (2013). Physiological and psychosocial age-related changes associated with reduced food intake in older persons. Ageing Res. Rev..

[31] Narendra D., Tanaka A., Suen D.F., Youle R.J. (2008). Parkin is recruited selectively to impaired mitochondria and promotes their autophagy. J. Cell Biol..

[32] Regmi S.G., Rolland S.G., Conradt B. (2014). Age-dependent changes in mitochondrial morphology and volume are not predictors of lifespan. Aging.

[33] Shen E.Z., Song C.Q., Lin Y., Zhang W.H., Su P.F., Liu W.Y., Zhang P., Xu J., Lin N., Zhan C. (2014). Mitoflash frequency in early adulthood predicts lifespan in *Caenorhabditis elegans*. Nature.

[34] Brennan J.P., Berry R.G., Baghai M., Duchen M.R., Shattock M.J. (2006). FCCP is cardioprotective at concentrations that cause mitochondrial oxidation without detectable depolarisation. Cardiovasc. Res..

[35] Gregg C., Kyryakov P., Titorenko V.I. (2009). Purification of mitochondria from yeast cells. J. Vis. Exp..

[36] Liao P.C., Bergamini C., Fato R., Pon L.A., Pallotti F. (2020). Isolation of mitochondria from cells and tissues. Methods Cell Biol..

[37] Dong M.Q., Venable J.D., Au N., Xu T., Park S.K., Cociorva D., Johnson J.R., Dillin A., Yates J.R. (2007). Quantitative mass spectrometry identifies insulin signaling targets in *C. elegans*. Science.

[38] Murphy C.T., McCarroll S.A., Bargmann C.I., Fraser A., Kamath R.S., Ahringer J., Li H., Kenyon C. (2003). Genes that act downstream of DAF-16 to influence the lifespan of Caenorhabditis elegans. Nature.

[39] Depuydt G., Xie F., Petyuk V.A., Smolders A., Brewer H.M., Camp D.G., Smith R.D., Braeckman B.P. (2014). LC-MS proteomics analysis of the insulin/IGF-1-deficient Caenorhabditis elegans daf-2(e1370) mutant reveals extensive restructuring of intermediary metabolism. J. Proteome Res..

[40] McElwee J.J., Schuster E., Blanc E., Thornton J., Gems D. (2006). Diapause-associated metabolic traits reiterated in long-lived daf-2 mutants in the nematode *Caenorhabditis elegans*. Mech. Ageing Dev..

[41] D’Antona G., Ragni M., Cardile A., Tedesco L., Dossena M., Bruttini F., Caliaro F., Corsetti G., Bottinelli R., Carruba M.O. (2010). Branched-chain amino acid supplementation promotes survival and supports cardiac and skeletal muscle mitochondrial biogenesis in middle-aged mice. Cell Metab..

[42] Edwards C., Canfield J., Copes N., Brito A., Rehan M., Lipps D., Brunquell J., Westerheide S.D., Bradshaw P.C. (2015). Mechanisms of amino acid-mediated lifespan extension in *Caenorhabditis elegans*. BMC Genet..

[43] Mansfeld J., Urban N., Priebe S., Groth M., Frahm C., Hartmann N., Gebauer J., Ravichandran M., Dommaschk A., Schmeisser S. (2015). Branched-chain amino acid catabolism is a conserved regulator of physiological ageing. Nat. Commun..

[44] Wodrich A.P.K., Scott A.W., Shukla A.K., Harris B.T., Giniger E. (2022). The Unfolded Protein Responses in Health, Aging, and Neurodegeneration: Recent Advances and Future Considerations. Front. Mol. Neurosci..

[45] Kikis E.A., Gidalevitz T., Morimoto R.I. (2010). Protein homeostasis in models of aging and age-related conformational disease. Adv. Exp. Med. Biol..

[46] Heintz C., Doktor T.K., Lanjuin A., Escoubas C., Zhang Y., Weir H.J., Dutta S., Silva-Garcia C.G., Bruun G.H., Morantte I. (2017). Splicing factor 1 modulates dietary restriction and TORC1 pathway longevity in *C. elegans*. Nature.

[47] Stout G.J., Stigter E.C., Essers P.B., Mulder K.W., Kolkman A., Snijders D.S., van den Broek N.J., Betist M.C., Korswagen H.C., Macinnes A.W. (2013). Insulin/IGF-1-mediated longevity is marked by reduced protein metabolism. Mol. Syst. Biol..

[48] Shin E.Y., Park J.H., You S.T., Lee C.S., Won S.Y., Park J.J., Kim H.B., Shim J., Soung N.K., Lee O.J. (2020). Integrin-mediated adhesions in regulation of cellular senescence. Sci. Adv..

[49] Lanza I.R., Nair K.S. (2010). Mitochondrial function as a determinant of life span. Pflügers Arch..

[50] Wallace D.C. (2010). Mitochondrial DNA mutations in disease and aging. Environ. Mol. Mutagen..

[51] Lee H.C., Wei Y.H. (2012). Mitochondria and aging. Adv. Exp. Med. Biol..

[52] Cai L., Sutter B.M., Li B., Tu B.P. (2011). Acetyl-CoA induces cell growth and proliferation by promoting the acetylation of histones at growth genes. Mol. Cell.

[53] Nisoli E., Clementi E., Moncada S., Carruba M.O. (2004). Mitochondrial biogenesis as a cellular signaling framework. Biochem. Pharmacol..

[54] Ren J., Pulakat L., Whaley-Connell A., Sowers J.R. (2010). Mitochondrial biogenesis in the metabolic syndrome and cardiovascular disease. J. Mol. Med..

[55] Kimura K.D., Tissenbaum H.A., Liu Y., Ruvkun G. (1997). daf-2, an insulin receptor-like gene that regulates longevity and diapause in *Caenorhabditis elegans*. Science.

[56] Butler J.A., Mishur R.J., Bhaskaran S., Rea S.L. (2013). A metabolic signature for long life in the *Caenorhabditis elegans* Mit mutants. Aging Cell.

[57] Saxton R.A., Sabatini D.M. (2017). mTOR Signaling in Growth, Metabolism, and Disease. Cell.

[58] Johnson S.C., Rabinovitch P.S., Kaeberlein M. (2013). mTOR is a key modulator of ageing and age-related disease. Nature.

[59] Burkewitz K., Zhang Y., Mair W.B. (2014). AMPK at the nexus of energetics and aging. Cell Metab..

[60] Giorgi C., Marchi S., Simoes I.C.M., Ren Z., Morciano G., Perrone M., Patalas-Krawczyk P., Borchard S., Jedrak P., Pierzynowska K. (2018). Mitochondria and Reactive Oxygen Species in Aging and Age-Related Diseases. Int. Rev. Cell Mol. Biol..

[61] Mitchell S.J., Bernier M., Mattison J.A., Aon M.A., Kaiser T.A., Anson R.M., Ikeno Y., Anderson R.M., Ingram D.K., de Cabo R. (2019). Daily Fasting Improves Health and Survival in Male Mice Independent of Diet Composition and Calories. Cell Metab..

[62] Houtkooper R.H., Mouchiroud L., Ryu D., Moullan N., Katsyuba E., Knott G., Williams R.W., Auwerx J. (2013). Mitonuclear protein imbalance as a conserved longevity mechanism. Nature.

[63] Covarrubias A.J., Perrone R., Grozio A., Verdin E. (2021). NAD(+) metabolism and its roles in cellular processes during ageing. Nat. Rev. Mol. Cell Biol..

[64] Korovila I., Hugo M., Castro J.P., Weber D., Hohn A., Grune T., Jung T. (2017). Proteostasis, oxidative stress and aging. Redox Biol..

[65] Ross J.M., Olson L., Coppotelli G. (2024). Mitochondrial Dysfunction and Protein Homeostasis in Aging: Insights from a Premature-Aging Mouse Model. Biomolecules.

[66] Tohyama D., Yamaguchi A., Yamashita T. (2008). Inhibition of a eukaryotic initiation factor (eIF2Bdelta/F11A3.2) during adulthood extends lifespan in *Caenorhabditis elegans*. FASEB J..

[67] Watts J.L., Ristow M. (2017). Lipid and Carbohydrate Metabolism in *Caenorhabditis elegans*. Genetics.

[68] Wang X., Yu H., Gao R., Liu M., Xie W. (2023). A comprehensive review of the family of very-long-chain fatty acid elongases: Structure, function, and implications in physiology and pathology. Eur. J. Med. Res..

