# Supplementary material for: Mitochondrial Proteome Reveals Metabolic Tuning by Restricted Insulin Signaling to Promote Longevity in Caenorhabditis elegans
Source: Biology (Basel). 2025 Mar 9;14(3):279. doi: 10.3390/biology14030279 (PMC11940386; doi:10.3390/biology14030279)
Supplement: Supplementary file 1 [file biology-14-00279-s001.zip › biology-3441751-supplementary figures.pdf]

Supplementary Materials:

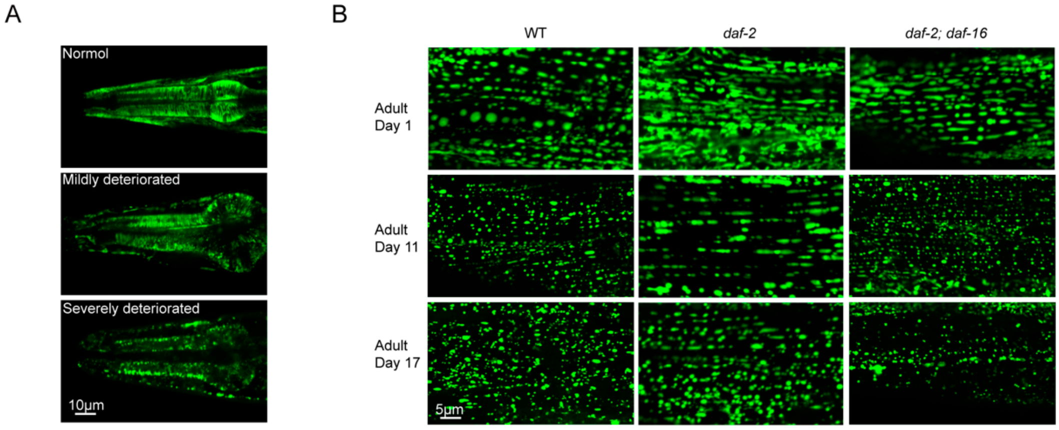

Figure S1. Age-dependent mitochondrial function decline was attenuated by mutation of *daf-2*. (**A**) Representative images of normal, mildly deteriorated, and severely deteriorated mitochondrial morphology in the pharynx. Normal: Mitochondria exhibit continuous filamentous or network-like structures with uniform distribution. Mildly Deteriorated: Some mitochondria show fragmentation and reduced length, but the overall network structure is still recognizable. Severely Deteriorated: Mitochondria are completely fragmented, forming numerous punctate fragments or highly aggregated granular structures. And we have provided an explanation in the revised version. (**B**) Representative images of mitochondrial morphologies in body wall muscle of WT, *daf-2, daf-2; daf-16* at days 1, 11, and 17.

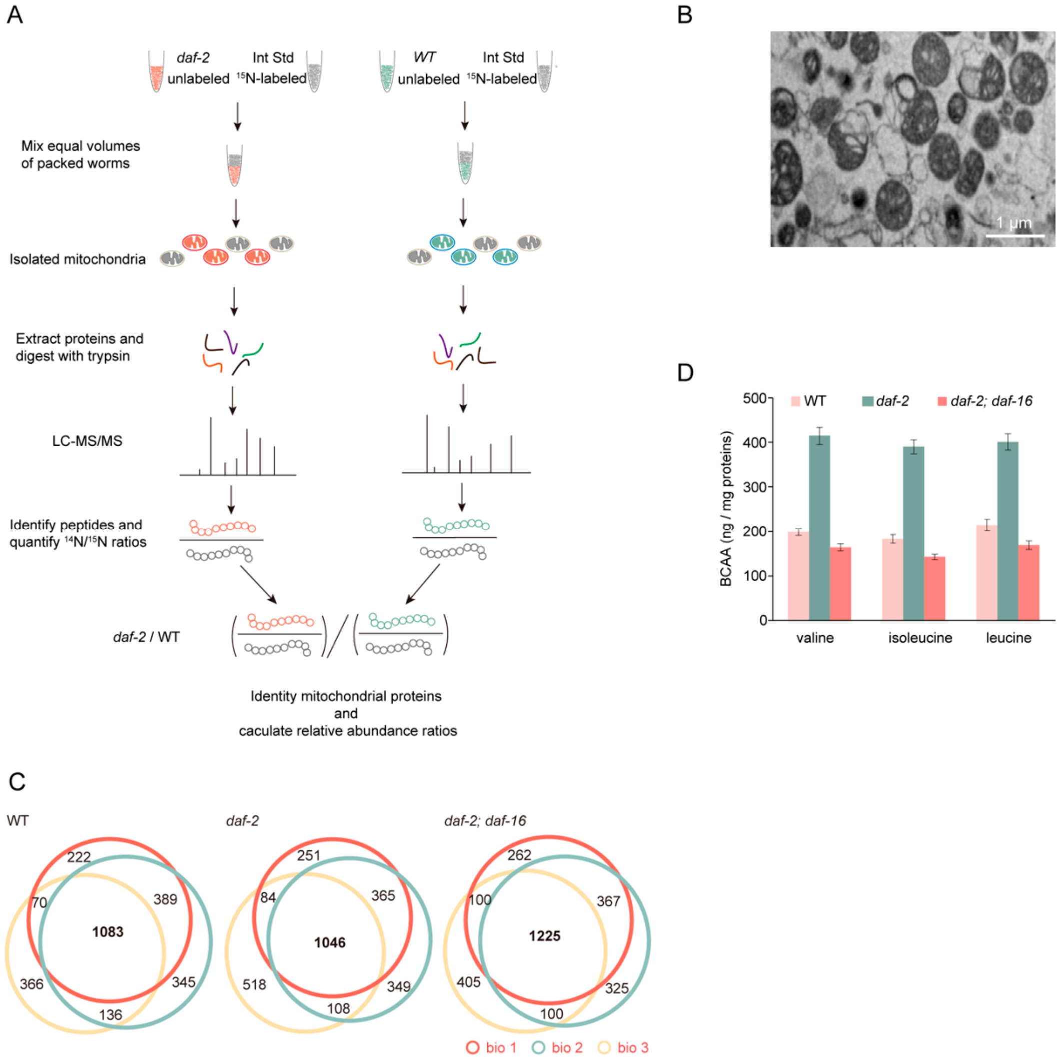

Figure S2. Quantitative mass spectrometry analysis of mitochondrial in WT, *daf-2* and *daf-2; daf-16* worms. (**A**) Workflow of mitochondria quantitative proteomic analysis. Int Std: internal standard. (**B**) Transmission electron microscopy (TEM) images of isolated mitochondria. (**C**) Overlap of identified proteins across biological replicates of mitochondrial samples from WT, *daf-2* mutant, *daf-2; daf-16* mutant. (**D**) The abundance of Valine, Isoleucine, and Leucine in WT, *daf-2* mutant, and *daf-2; daf-16* mutant showed by absolute quantification..

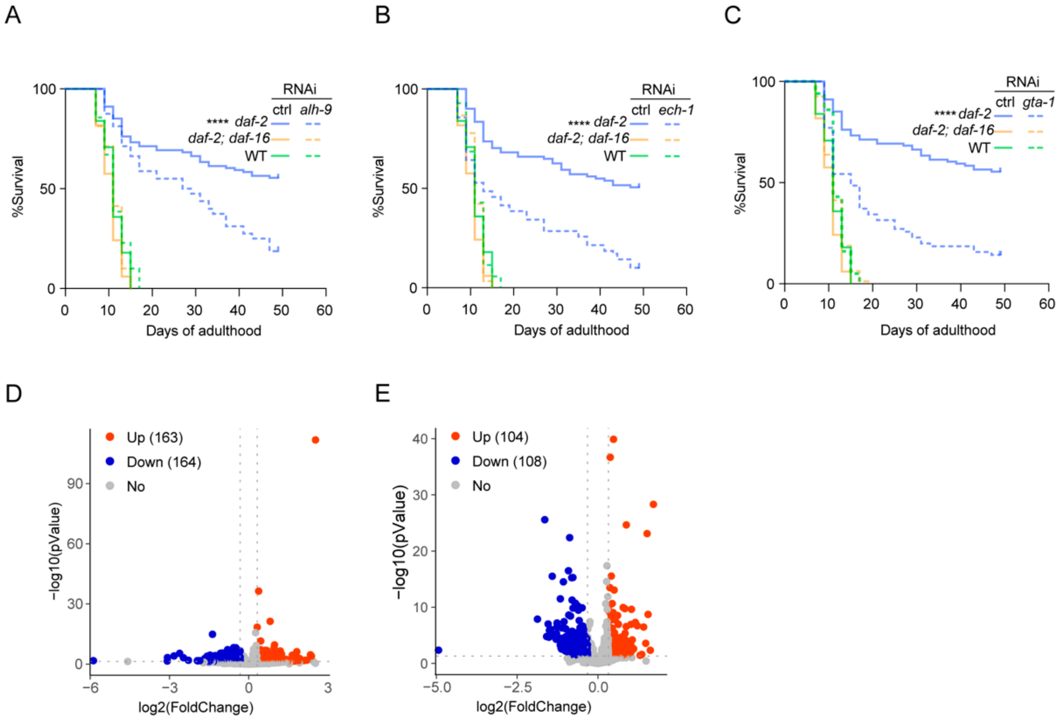

Figure S3. Lifespan and mRNA-seq analysis of *daf-2 (e1370)* worms treated by RNAi of three metabolic enzymes. (**A-C**) Lifespan analysis of RNAi-treated WT, *daf-2*, and *daf-2; daf-16* worms. (A) *alh-9* RNAi. (D) *ech-1* RNAi. (C) *gta-1* RNAi. (**D**-**E**) Volcano plot of differentially expressed genes (DEGs) of WT, *daf-2*, *daf-2; daf-16* mutants treated with RNAi. (D) *cpt-2* RNAi treatment. (E) *ech-4* treatment. (∣Fold-change∣ > 1.25, FDR<0.05).

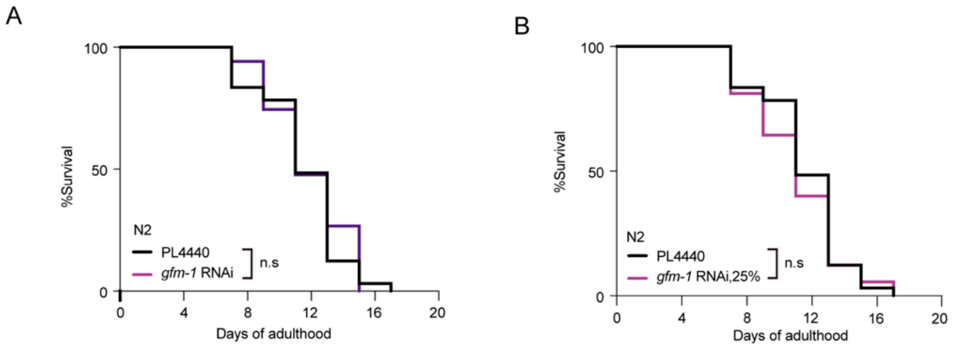

Figure S4. Lifespan analysis following RNAi knockdown of GFM-1 in WT, *daf-2*, and *daf-2*; *daf-16* mutants with (**B**) or without (**A**) RNAi dilution.
